# Treatment of complex urethral stenosis in public centers from developing countries in 21st century

**DOI:** 10.1590/S1677-5538.IBJU.2021.99.16

**Published:** 2021-06-15

**Authors:** Silvio Tucci, Henrique Donizetti Bianchi Florindo

**Affiliations:** 1 Universidade de São Paulo – USP-Ribeirão Preto Faculdade de Medicina de Ribeirão Preto Divisão de Urologia Ribeirão Preto SP Brasil Divisão de Urologia, Faculdade de Medicina de Ribeirão Preto, Universidade de São Paulo – USP-Ribeirão Preto, Ribeirão Preto, SP, Brasil

## COMMENT

Access to surgical treatment may be the only solution for preventing chronic disabilities and mortality (1) in cases of traumatic injuries and countless other situations that significantly affect quality of life such as iatrogenic, inflammatory or neoplastic injury. Notwithstanding successful prevention strategies, these conditions are responsible for a significant portion of disease burden in the population. In practice, speedy access to essential surgery is not always widely guaranteed (2), although it is part of the human right to health. In developing countries, where the most appropriate treatment for various diseases is not always readily available (2), there is a high incidence of complications from delays in diagnosis or access to specialized centers. In Brazil, this is the context in which complex urethral stenosis in public university institutions linked to the Unified Health System is approached.

Male urethral stenosis has significant negative impact on patients’ quality of life. Its pathophysiology is better known and differences between developed and developing countries must be considered. The recent study by Astolfi et al, (3) evaluating 899 patients, shows that in Brazil iatrogenesis was the most frequent cause (43.2%), followed by idiopathic (21.7%) and traumatic (21.5%). Of the inflammatory causes (13.7%), the largest part was due to scleroatrophic lichen (66.7%), and 33.4% to infectious urethritis.

Regarding the stenosis site, Palminteri et al. (4) showed that in developed countries, stenosis occurs mainly in the anterior urethra (92.2%), particularly in the bulbar segment (45.9%), with involvement of the posterior urethra in only 7.8% of cases (5). Most trauma-related stenosis occurred in urethral lesions associated with pelvic fractures (62.7%) and 62.7% were associated with perineal trauma. Of iatrogenic causes, 59% were secondary to urethral instrumentation (catheterization and other procedures), 24.8% due to procedures such as prostatectomy, radiotherapy and postectomy, and 16.2% following failure to correct hypospadias.

Such demographic data are useful in guiding the development of preventive and therapeutic population strategies, as well as being important for educational guidance in relation to manipulation of the urethra (6), from vesicoureteral catheterization to instrumentation of the urethra in therapeutic procedures and, above all, to learn the different urethroplasty techniques. Strategies must be guided by the Unified Health System (SUS) as set forth in Article 5 of Law 8.080 of September 1990, and educational guidance is an obligation of medical schools and specific courses in Urology as a specialty.

The SUS offers universal coverage and free access to all levels of health care for each person within its territory, but there may be long waiting periods for exams or surgery. Tertiary care centers and the best health care services are unevenly concentrated in the richest regions of the country, making access more difficult for the population living in more disadvantaged areas. In order to reduce the impact of such inequality, Out-of-Home Treatment was established by Ordinance No. 55 of the Ministry of Health, an instrument that aims, through the SUS, to guarantee medical treatment to patients with diseases that are untreatable in their municipality of origin due to lack of technical conditions.

Although SUS clinical care and treatment strategies are well organized, with specific programs for different diseases, in the case of urethral stenosis the current situation has space for improvement. Patients still go through various difficulties to reach referral centers, resulting in delayed access to definitive treatment. This can worsen prognosis due to episodes of urinary tract infection and secondary changes in the upper urinary tract. At the same time, doctors who provide initial care and those at the Reference Centers face their own difficulties.

Not every urologist is able to perform surgical correction of the various forms of urethral stenosis, whether in adults or children. Referrals for the most classic techniques, such as urethral dilatations and urethrotomies, are much more selective today, although they are still widely performed. Without appropriate referral, they provide only temporary resolution and often increase the extent of the primary lesion (7). In other countries, developed or otherwise, these techniques are still performed by the majority of practicing urologists (8–11) in spite of there being excellent centers where all modern modalities are performed.

Nowadays, urethroplasties require years of study to obtain the most lasting results, and some authors consider proficiency as occurring after around 100 surgeries (12). Thus, it may be that urethroplasties are underutilized due to lack of training or access to a trained colleague (13, 14). It is important to consider, then, that training in reconstructive urology should be encouraged and offered by the various Medical Residency Services in Urology in Brazil (15, 16).

Depending on the etiological factor or the extent of the urethral lesion (17, 18), some patients with urethral stenosis experience complications such as erectile dysfunction and/or urinary incontinence, requiring additional procedures, such as implantation of penile prostheses and artificial sphincters, which are generally costly and are not always widely available in the public health care services.

We highlight the importance of greater speed in referring patients to SUS Reference Centers where professionals trained to perform the different types of urethroplasties practice, as there is a certain dispute due to supply and demand ratio of slots available to the municipalities in their scope. The Municipality schedules the consultation, but scheduling slots should not be undertaken by an administrative officer who is unfamiliar with the functional implications of each disease. It should be undertaken by a doctor able to differentiate the more serious situations, using the available slots correctly.

In reference public health care centers, there are generally several specialties operating, each with a historical queue of patients awaiting surgical treatment and, as a result, operating rooms have different distributions on weekdays. In Urology, there are other important diseases such as tumors, urinary obstructions due to other causes, anomalies, lithiasis, etc. Thus, in addition to the availability of slots for surgery, there is also internal competition for use of operating rooms between the more serious diseases. Another fact to be considered is the lack of counter-referral for following up complex cases after treatment; the impossibility of outpatient visits at a longer time interval reduces the number of places available for new patients.

Urological knowledge advances quickly and requires constant updating, in addition to careful analysis of international literature. The national guidelines need updating to adapt to the current context of urological work in public centers in developing countries, aiming to reconcile information from the medical field, in order to standardize behavior that supports reasoning and decision-making, which must be subjected to their own assessment and criticism, given the reality and clinical status of each patient.

With regard to trauma, in particular, in public hospitals Brazilian urologists, in most cases, do not have access to the instruments that would help them provide the best emergency care; the rigid cystoscope in general is not a reality in SUS emergency cases, and the flexible cystoscope is not available even in private hospitals. Often, retrograde urethrocystography itself (a low cost, easy to perform and accurate test, which allows for better treatment planning) (19) cannot be offered.

Traumatic urethral injuries are not uncommon, particularly in severely injured patients (17, 18). Supra-pubic urinary diversion is often performed in trauma in critically ill patients to enable accurate monitoring of urine output and resuscitation. Immediate surgical repair can be technically difficult and should be limited to patients who experience penetrating urethral trauma and are hemodynamically stable. Injuries to the posterior urethra are often associated with major abdominal trauma or pelvic fractures that are life-threatening and require immediate attention and resuscitation. Anterior urethral injuries are usually the result of direct trauma to the urethra, with few associated and less extensive injuries (6).

Incomplete posterior urethral lesions can be treated with bladder catheterization, however, there is a risk of it turning into a complete lesion, where treatment options are immediate primary surgical reconstruction (currently abandoned due to high rates of erectile dysfunction, incontinence, stenosis and intraoperative bleeding), primary realignment in stable patients (which decreases rates of stenosis and incontinence and is the choice in several referral services) or supra-pubic urinary diversion and late reconstruction (classic and often mandatory management due to the clinical instability of polytrauma patients) (6, 12), with urethral stenosis as the rule in the latter case. Late posterior perineal urethroplasty has high success rates and avoids the need for multiple procedures (17, 18).

Erectile dysfunction occurs in approximately 50% of patients, has a multifactorial etiology (and results from trauma aggression, not treatment). In patients undergoing posterior urethroplasty, 5 to 15% will develop recurrent stenosis and about 4% to 10% will develop urinary incontinence (16, 17). These complications and functional sequelae are also treated by the SUS, but not always in the same proportion as they occur. It is in this sphere that the greatest difficulties are concentrated for professionals who work with reconstructive urology in the public health care service, much more than those related to the techniques of urethroplasty itself. Access to penile prostheses and artificial sphincters is limited to a few services in Brazil. Moreover, the functional impact resulting from postponed treatment affects surgical procedures and referrals.

Narrowing in the penile urethra is less frequent, however, it tends to be longer than those present in the bulbar urethra. Due to the narrowness of the cancellous body and, consequently, greater spongiofibrosis, endoscopic treatment has lower success rates and should not be routinely indicated, being reserved only for ring stenoses (7, 8). Excision techniques and primary anastomosis of the penile urethra result in penile curvature or shortening due to contact with the corpora cavernosa. Thus, the treatment of choice is substitute urethroplasty (graft or flap), or staged urethroplasty in more complex cases. Use of foreskin flaps should be considered as they are more accessible in the penile urethra, decreasing morbidity and surgical time, however, success rates are lower than for use of oral mucosa, which is widely used due to excellent success rates; the graft is preferably positioned on the dorsal surface of the urethra (6).

As for narrowing of the bulbar urethra, when short (up to 2cm), of idiopathic cause and with little spongiofibrosis, it can be treated with endoscopic urethrotomy under direct vision (8), more modern methods of internal urethrotomy with laser have good success rates (70 to 80%), however, limited to a single approach in patients who have not had other treatment and to short impairments, due to the high rate of recurrence (20).

Bulb narrowing of traumatic or inflammatory origin leads to extensive spongiofibrosis. Initially, the plan is to remove spongiofibrosis whenever possible and perform wide spatulated anastomosis between two healthy stumps, however, due to the risk of penile curvature or shortening, excision and primary anastomosis are limited to up to 3cm (6, 8).

In narrowing where obliteration of the urethral lumen is not complete, with involvement exceeding 3cm and of non-traumatic etiology, substitute techniques can be used with oral mucosa graft, with graft located in the dorsal or ventral region or in both regions, with similar results. In more complex cases, enlarged urethroplasty techniques can be used, with resection and mucosa graft (6).

Evidently, there are no simple solutions to a situation as complex as health care in a country like Brazil, the size of a continent and with great regional diversity. The immense effort the Unified Health System makes to facilitate the population's access to health care, in its most diverse areas, is praiseworthy, and seeking to improve this program, in order to comply with Article 196 of the Constitution, must be ongoing on the part of responsible authorities. There is much to be done. Public institutions linked to the SUS, in their turn, also make great efforts to operate as an effective part of this System.

As we have seen, there are countless challenges in the treatment of urethral stenosis in Public Health Care Centers nowadays. In this context, the role of teaching hospitals transcends the sphere of continuing education, by influencing our conduct and the doctor-patient relationship. Therefore, the choice of conduct and information transmitted to our patients must be an absolute priority in our daily practice. Even in the face of all these challenges, urologists who work with reconstructive surgery feel stimulated in the face of the complexity of the cases and combat adversity by remaining motivated to do, above all, what they were trained as doctors to do: comfort, relieve and treat the sick.
